# Disruption of the Circadian Clock Alters Antioxidative Defense via the SIRT1-BMAL1 Pathway in 6-OHDA-Induced Models of Parkinson's Disease

**DOI:** 10.1155/2018/4854732

**Published:** 2018-04-18

**Authors:** Yali Wang, Dongjun Lv, Wenwen Liu, Siyue Li, Jing Chen, Yun Shen, Fen Wang, Li-Fang Hu, Chun-Feng Liu

**Affiliations:** ^1^Department of Neurology, Suzhou Municipal Hospital, Nanjing Medical University, Suzhou 215008, China; ^2^Department of Neurology and Suzhou Clinical Research Center of Neurological Disease, The Second Affiliated Hospital of Soochow University, Suzhou 215004, China; ^3^Jiangsu Key Laboratory of Neuropsychiatric Diseases and Institute of Neuroscience, Soochow University, Suzhou 215123, China

## Abstract

Parkinson's disease (PD) is the second most common neurodegenerative disease and is known to involve circadian dysfunction and oxidative stress. Although antioxidative defense is regulated by the molecular circadian clock, few studies have examined their function in PD and their regulation by silent information regulator 1 (SIRT1). We hypothesize that reduced antioxidative activity in models of PD results from dysfunction of the molecular circadian clock via the SIRT1 pathway. We treated rats and SH-SY5Y cells with 6-hydroxydopamine (6-OHDA) and measured the expression of core circadian clock and associated nuclear receptor genes using real-time quantitative PCR as well as levels of SIRT1, brain and muscle Arnt-like protein 1 (BMAL1), and acetylated BMAL1 using Western blotting. We found that 6-OHDA treatment altered the expression patterns of clock and antioxidative molecules *in vivo* and *in vitro*. We also detected an increased ratio of acetylated BMAL1:BMAL1 and a decreased level of SIRT1. Furthermore, resveratrol, an activator of SIRT1, decreased the acetylation of BMAL1 and inhibited its binding with *CRY1*, thereby reversing the impaired antioxidative activity induced by 6-OHDA. These results suggest that a dysfunctional circadian clock contributes to an abnormal antioxidative response in PD via a SIRT1-dependent BMAL1 pathway.

## 1. Introduction

Parkinson's disease (PD) is the second most common neurodegenerative disease, affecting about 1.7% of people over 65 years of age [1]. PD is characterized by the loss of dopaminergic neurons in the substantia nigra pars compacta and the formation of Lewy bodies. Many clinical manifestations of PD hint at a dysfunction of circadian rhythm [2], including disturbed rest-activity rhythm, sleep disorders, an abnormal daily pattern of blood pressure, and altered secretion of melatonin. Furthermore, the expression of circadian clock molecules is altered in PD patients and animal models of PD [3, 4].

The circadian system exists in virtually all organisms and serves to coordinate behavior and physiology with the surrounding environment [5]. At the molecular level, the circadian system mainly consists of a set of conserved clock proteins, including brain and muscle Arnt-like protein 1 (BMAL1), clock (CLOCK), period circadian protein (PER1–3), and cryptochrome (CRY1,2). Of these, the heterodimer of BMAL1 and CLOCK drives transcription of a host of genes referred to as clock-controlled genes [6]. Interestingly, antioxidative genes exhibit daily rhythms in their expression and activity in different brain regions, indicating that they are under the endogenous control of the circadian system [7, 8]. Also, clock-responsive elements (E-box or E-box-like) are found in the promoter regions of antioxidative genes. Furthermore, animals deficient in clock proteins show increased reactive oxygen species generation and decreased antioxidative enzyme activity [9–11].

The pathology of PD is complicated and multifactorial, involving oxidative stress, neuroinflammation, mitochondrial impairment, and impaired protein degradation. Depletion of endogenous antioxidants and increased oxidative stress are thought to play a particularly important role in the progression of PD [12]. The expression and activity of catalase (CAT), superoxide dismutase (SOD), and glutathione peroxidase (GPx) are attenuated in neurotoxin-induced animal models of PD. And the reactive oxygen species (ROS) and lipid peroxide (LPO) generation are induced by the neurotoxin [13–15]. Postmortem examination of PD patients reveals reduced glutathione (GSH) content in the substantia nigra [15]. In addition, the mutation of DJ-1, which plays an important role in antioxidative defense, is a cause of familial PD [16]. Considering these findings, the reestablishment of redox homeostasis is a potential therapeutic strategy for sporadic and familial PD.

Silent information regulator 1 (SIRT1) is a member of the sirtuin family of proteins, which are highly conserved aldehyde dehydrogenase-dependent class III deacetylases [17]. Gene polymorphism in the *SIRT1* gene promoter, which could repress its transcription, may be a risk factor for PD [18]. In a PD model, SIRT1 protein is reduced, and SIRT1 agonist exerts a protective effect against PD [19]. Importantly, SIRT1 affects circadian rhythm and the expression of clock-controlled genes by deacetylating BMAL1 and PER2 proteins and binding with the CLOCK-BMAL1 complex [20, 21]. In chronic obstructive pulmonary disease, the SIRT1-BMAL1 pathway mediates the regulatory effect of circadian dysfunction on the inflammatory response [22]. Similarly, the patterns of antioxidative gene expression also display circadian oscillations. As BMAL1 is decreased in PD [3], it is likely that the circadian dysfunction in PD could contribute to the altered expression of antioxidative genes. Nevertheless, research on this possibility and its underlying mechanism is currently lacking. Here, we aimed to verify whether molecular circadian clock function and antioxidative defense are disrupted in PD and to investigate whether the SIRT1-BMAL1 pathway is involved in the abnormal expression of antioxidative genes in PD.

## 2. Materials and Methods

### 2.1. Reagents and Antibodies

6-Hydroxydopamine (6-OHDA) and resveratrol (R5010) were purchased from Sigma-Aldrich (St. Louis, MO, USA). Primary antibodies against BMAL1, acetylated BMAL1, were obtained from Abcam (Cambridge, MA, USA) and Millipore (Billerica, MA, USA), and antibodies against SIRT1 and CRY1 were bought from Santa Cruz Biotechnology (Dallas, TX, USA). Reagents for cell culture were bought from HyClone (GE Healthcare, Buckinghamshire, UK). Kits for ROS (reactive oxygen species) and LPO (lipid peroxide) were obtained from Jiancheng Biotechnology Company (Nanjing, China).

### 2.2. Animal Model of PD Induced by 6-OHDA

Thirty Sprague-Dawley male rats (180–220 g) were obtained from Slac Laboratory Animal Company (Shanghai, China). Rats were maintained on a 12:12 light:dark cycle with lights on at 8:00 and free access to food and water. All experimental procedures were performed according to the guidelines of the University Committee on Animal Care of Soochow University. Rats were randomly divided into two groups that received bilateral stereotaxic injections of either 6-OHDA or saline into the striatum. Briefly, after rats were anesthetized by chloral hydrate, injections were performed using a stereotaxic apparatus (David Kopf Instruments, Tujunga, CA, USA) with a 10 *μ*L Hamilton syringe. Lesions were induced by injection of 6-OHDA (2 *μ*g 6-OHDA in 1 *μ*L 0.02% ascorbic acid saline solution) into the striatum bilaterally (8 *μ*g 6-OHDA at each site) at the following coordinates: AP, +1.0; ML, ±3.0; DV, 4.5 mm. Three weeks later, behavioral tests were conducted to identify unsuccessful models, which were removed from the study. Another 3 weeks later, striatum tissue was collected at 6:00, 12:00, 18:00, or 24:00 under dim red light.

### 2.3. SH-SY5Y Cell Culture and Treatment

SH-SY5Y cells were cultured in high-glucose Dulbecco's modified Eagle's medium with 10% fetal bovine serum and 1% penicillin and streptomycin in an atmosphere of 5% CO_2_ and 95% O_2_ at 37°C. All experiments were performed after 3–10 passages when cells reached ∼80–90% confluence. Cells were treated with 10 nM dexamethasone to synchronize clock genes for 2 h, followed by 6-OHDA treatment.

### 2.4. Western Blotting

Tissue samples and cells were homogenized in lysis buffer (150 mM NaCl, 5 mM EDTA, 25 mM Tris, 1% Nonidet P-40, pH 7.5). Homogenates were centrifuged at 15,000*g* for 20 min at 4°C. Supernatants were collected, and protein concentrations were measured with a commercial BCA kit (Thermo Scientific, Waltham, MA, USA). Extracted protein was separated on 10% Tris-glycine gels and transferred onto polyvinylidene fluoride membranes (Millipore, Bedford, MA, USA). After blocking in 5% milk, membranes were incubated with primary antibodies at 4°C overnight. Membranes were then washed with Tris-buffered saline containing 0.1% (v/v) Tween 20 and incubated with horseradish peroxidase-conjugated secondary antibodies at room temperature for 1 hour. Finally, protein bands were visualized with a luminescence kit (Millipore) and detected with ChemiDoc XRS+ (Bio-Rad, Hercules, CA, USA). The final results were analyzed using ImageJ software.

### 2.5. Real-Time Quantitative PCR (qPCR)

Total RNA was extracted from tissue samples and cells by TRIzol reagent (Invitrogen, Camarillo, CA, USA) according to the manufacturer's instructions. Reverse transcription was performed using the cDNA synthesis kit (Roche Diagnostics GmbH, Mannheim, Germany). PCR reactions were conducted with the 7900HT Fast Real-Time PCR System (Applied Biosystems, Foster City, CA, USA). qPCR primers were as follows: r*18S* (forward: 5′-TCAACACGGGAAACCTCAC-3′, reverse: 5′-CGCTCCACCAACTAAGAAC-3′); r*Bmal1* (forward: 5′-CTTGTCTGTAAAACTTGCCTGTGAC-3′, reverse: 5′-GTAGATCAGAGGGCGACGGCTA-3′); r*Clock* (forward: 5′-GTAGGTTTCCAGTCCTGTCG-3′, reverse: 5′-TGGGGTCTATGCTTCCTGGT-3′); r*Per2*(forward: 5′-AGGATCCAAGAACGGCACAG-3′, reverse: 5′-CGGACCTGGCTTCAGTTCAT-3′); r*Rorα* (forward: 5′-CCAAACTTGACAGCATCTCGA-3′, reverse: 5′-GAAGGCTGCAAGGGCTTTTTCAGGA-3′); r*Sod* (forward: 5′-AGGATTAACTGAAGGCGAGCAT-3′, reverse: 5′-TCTACAGTTAGCAGGCCAGCAG-3′); r*Cat* (forward: 5′-ACGAGATGGCACACTTTGACAG-3′, reverse: 5′-TGGGTTTCTCTTCTGGCTATGG-3′); r*Gpx* (forward: 5′-AAGGTGCTGCTCATTGAGAATG-3′, reverse: 5′-GAAGGCTGCAAGGGCTTTTTCAGGA-3′); r*Gst* (forward: 5′-GCTGGAGTGGAGTTTGAAGAA-3′, reverse: 5′-GTCCTGACCACGTCAACATAG-3′); h*Bmal1* (forward: 5′-CTGGCTAGAGTGTATACGTTTGG-3′, reverse: 5′-GGTCACCTCAAAGCGATTTTC-3′); h*Clock* (forward: 5′-AAAATACTCTCTACTCATCTGCTGG-3′, reverse: 5′-ATGGCTCCTTTGGGTCTATTG-3′); h*Per2* (forward: 5′-GCCAGAGTCCAGATACCTTTAG-3′, reverse: 5′-TGTGTCCACTTTCGAAGACTG-3′); h*Rorα* (forward: 5′-CTAGCTCTTCAACACGTCCTAC-3′, reverse: 5′-TCGCACAATGTCTGGGTATATT-3′); h*Sod* (forward: 5′-AGGCCGTGTGCGTGCTGAAG-3′, reverse: 5′-CACCTTTGCCCAAGTCATCTGC-3′); h*Cat* (forward: 5′-CCTTTCTGTTGAAGATGCGGCG-3′, reverse: 5′-GGCGGTGAGTGTCAGGATAG-3′); h*Gpx* (forward: 5′-GTGTATGCCTTCTCGGCGCG-3′, reverse: 5′-CGTTGCGACACACCGGAGAC-3′); h*Gst* (forward: 5′-GATACTGGGGTACTGGGACATCC-3′, reverse: 5′-CCACTGGCTTCTGTCATAATCAGG-3′); h*Gadph* (forward: 5′-CATGTTCGTCATGGGTGTGAACCA-3′, reverse: 5′-AGTGATGGCATGGACTGTGGTCAT-3′). The final results were calculated using the 2^−∆∆Ct^ method.

#### 2.5.1. LPO Content Assay

Tissue samples were collected at 6:00, 12:00, 18:00, or 24:00 at 3 weeks after the 6-OHDA lesion. The tissue was homogenized and centrifuged at 4000*g* for 20 min, followed by collecting the supernatant for assay. LPO in tissue homogenate was measured according to the manufacture's manual. Tissue homogenate was mixed with agents in the kit then incubated at 45°C for 60 min. The mixture was centrifuged for 10 min in 4000*g*. Then optical density of the supernatant was measured at 586 nm. LPO content was calculated according to the standard curve, and the result was expressed as *μ*mol per gram protein.

#### 2.5.2. ROS Detection

2,7-Dichlorofluorescin diacetate (DCFH-DA) was widely used for ROS measurement, and it is quite sensitive. *In vivo*, the DCFH-DA was added into the medium and incubated at 37 for 60 min. The image can be taken by the fluorescence microscope under FITC mode. Then, the cell was digested by trypsin and resuspended in PBS.

Then, the cells were transferred into a 96-well plate. The plate was read on a microplate reader (excitation at 485 nm and emission at 525 nm), and the result was expressed as the ratio of fluorescence value.

### 2.6. Coimmunoprecipitation

Twenty-four hours after 6-OHDA treatment, cells were harvested, washed with cold PBS, and lysed with lysis buffer. Equal amounts of proteins were conjugated with anti-BMAL1 antibodies overnight at 4°C followed by incubation with protein A/G-agarose beads for 2 h at room temperature. The beads were then washed with PBS three times and eluted with loading buffer followed by Western blotting. Membranes were incubated with primary antibodies against CRY1 and BMAL1.

### 2.7. Statistical Analysis

Values are expressed as mean ± SEM. Daily variations in each parameter were analyzed using one-way ANOVA. The 6-OHDA-lesioned group was compared with the sham group at particular time points using Student's *t*-tests. Data from cell experiments were analyzed using one-way ANOVA with Turkey's post hoc tests. Differences were considered statistically significant when *P* value < 0.05.

## 3. Results

### 3.1. Altered Clock and Antioxidative Gene Expression in the Striatum of 6-OHDA-Lesioned Rats

**Figure 1:**
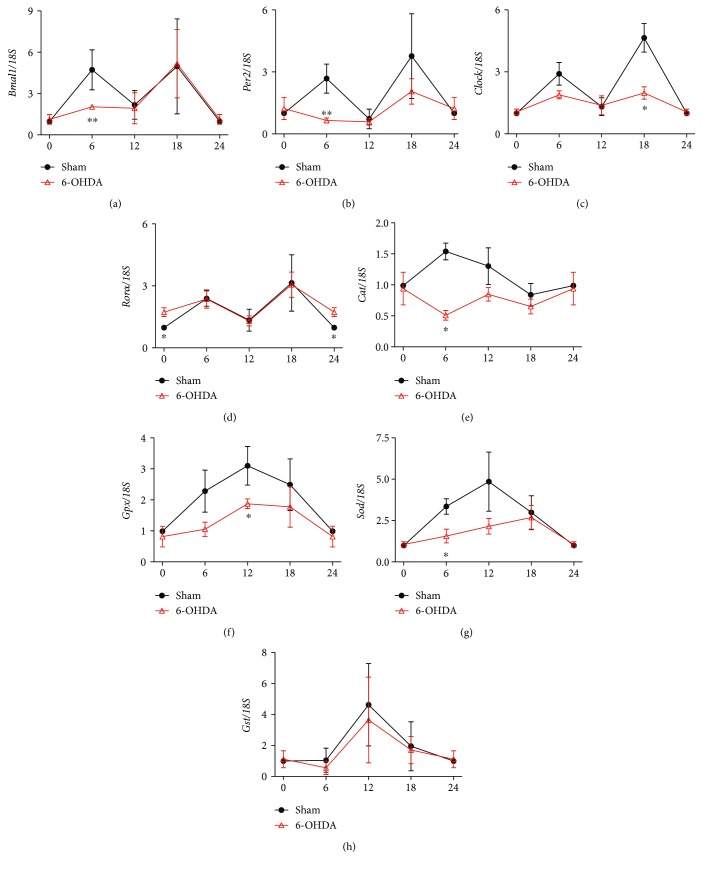
Clock and antioxidative gene expression was altered in the striatum of 6-OHDA-lesioned rats. (a) Expression of *Bmal1*. (b) Expression of *Per2*. (c) Expression of *Clock*. (d) Expression of *Rorα*. (e) Expression of *Cat*. (f) Expression of *Gpx*. (g) Expression of *Sod*. (h) Expression of *Gst*. *n* = 3–4 per time points (equals to *n* = 12 for each group), ^∗^*P* < 0.05 versus sham group, ^∗∗^*P* < 0.01.

Circadian rhythm plays an important role in regulating antioxidative activity. We used qPCR to measure clock and antioxidative gene expression in a 6-OHDA-lesioned animal model of PD. In the striatum of sham rats, mRNA levels of all genes except *Clock* showed significant circadian rhythms (*P* < 0.05) (Figures 1(a)–1(c)). However, mRNA levels of *Bmal1*, *Per2*, and *Clock* were decreased in 6-OHDA-lesioned rats compared with sham rats, especially at 6:00 and 18:00. By contrast, the mRNA level of retinoid acid receptor-related orphan receptor *α* (*Rorα*), which is a positive regulator of *Bmal1* expression, was increased in 6-OHDA-lesioned rats (Figure 1(d)). GPX, SOD, CAT, and glutathione S transferase (GST) are important enzymes that defend against oxidative stress. In 6-OHDA-lesioned rats, *Cat* and *Gpx* mRNA levels were decreased, although there were no obvious alterations in their circadian rhythm (Figures 1(e) and 1(f)). The mRNA level of *Sod* also declined and showed altered circadian rhythm, with a shift in its peak from 6:00 to 18:00 (Figure 1(g)). However, 6-OHDA had no effect on the mRNA level of *Gst* (Figure 1(h)).

### 3.2. Decreased Clock and Antioxidative Gene Expression in 6-OHDA-Treated SH-SY5Y Cells

**Figure 2:**
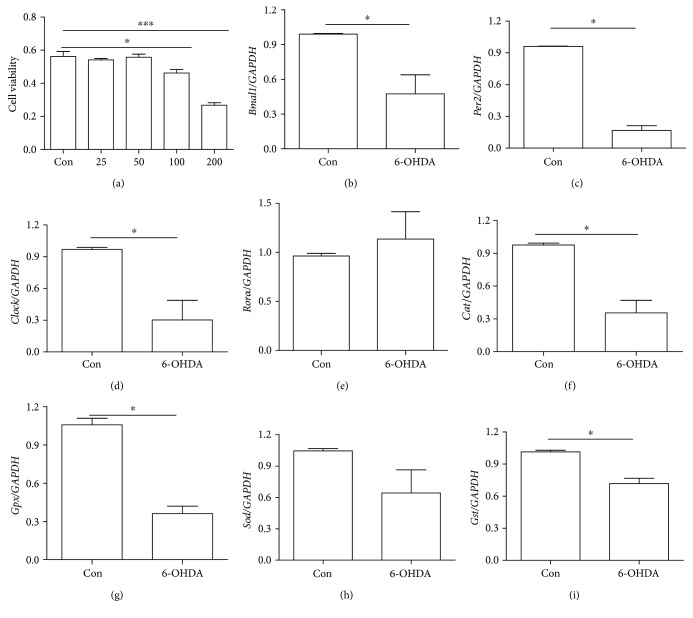
Clock and antioxidative gene expression was decreased in 6-OHDA-treated SH-SY5Y cells. (a) Cell viability after 6-OHDA treatment. (b) Expression of *Bmal1*. (c) Expression of *Per2*. (d) Expression of *Clock*. (e) Expression of *Rorα*. (f) Expression of *Cat*. (g) Expression of *Gpx*. (h) Expression of *Sod*. (i) Expression of *Gst*. *n* = 3 per group, ^∗^*P* < 0.05 versus control group, ^∗∗∗^*P* < 0.001 versus control group.

6-OHDA-treated SH-SY5Y cells are a classic *in vitro* model of PD. We observed no significant decline in cell viability after 0–100 *μ*M 6-OHDA treatment and over 50% cell death after 200 *μ*M 6-OHDA treatment (Figure 2(a)). Therefore, we used 100 *μ*M 6-OHDA to evaluate its effect on the mRNA levels of clock and antioxidative genes. Compared with control cells, 6-OHDA-treated cells showed decreased mRNA levels of *Bmal1*, *Clock*, *Per2*, and *Rorα* (Figures 2(b)–2(e)). In addition, mRNA levels of the antioxidative genes *Cat*, *Gpx*, and *Gst* were decreased in 6-OHDA-treated cells, and there was a trend toward a decrease in *Sod* mRNA level (Figures 2(f)–2(i)).

### 3.3. LPO and ROS Were Decreased in 6-OHDA-Treated Rat and Cell Model

**Figure 3:**
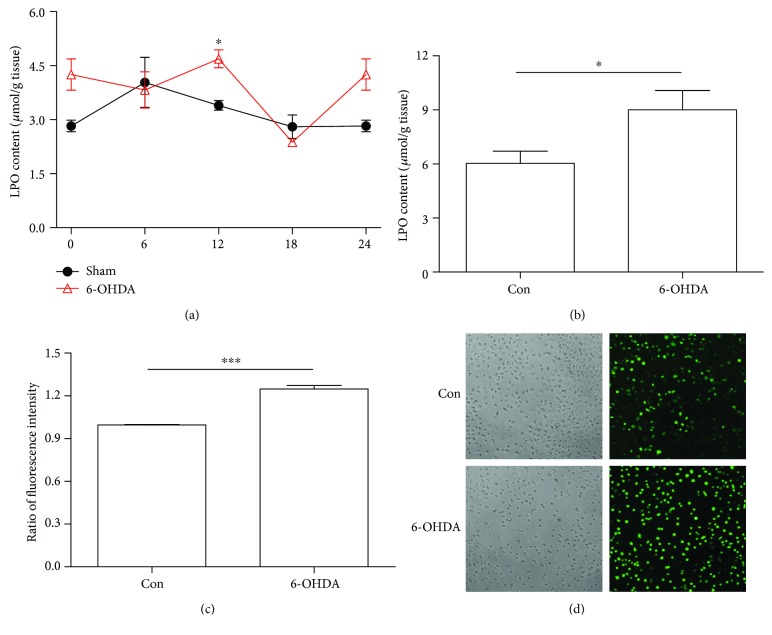
LPO and ROS were increased in 6-OHDA-treated rat and cell model. (a) LPO content in 6-OHDA-treated rat model. (b) LPO content in 6-OHDA-treated cell model. (c) The ratio of fluorescence intensity in cell model. (d) The representative pictures were shown, and the green spots were for ROS (20x). ^∗^*P* < 0.05 versus control group, ^∗∗∗^*P* < 0.001 versus control group.

LPO and ROS are the classical indexes of oxidative stress. Here, we have conducted experiments to detect the LPO level *in vivo* and *in vitro*. In the striatum of sham rats, the LPO contents showed significant circadian rhythms (one-way ANOVA, *P* < 0.05). However, it was increased in 6-OHDA-lesioned rats compared with sham rats, especially at 12:00 (Figure 3(a)). Consistently, in cell model, 6-OHDA increased levels of LPO by about 30% when compared with control group (Figure 3(b)). In addition, we also observed the ROS content *in vivo*. 6-OHDA treatment could induce the ROS production by 20% (Figure 3(c)). The representative pictures were shown in Figure 3(d).

### 3.4. Altered Acetylated BMAL1 and SIRT1 Levels after 6-OHDA Treatment

**Figure 4:**
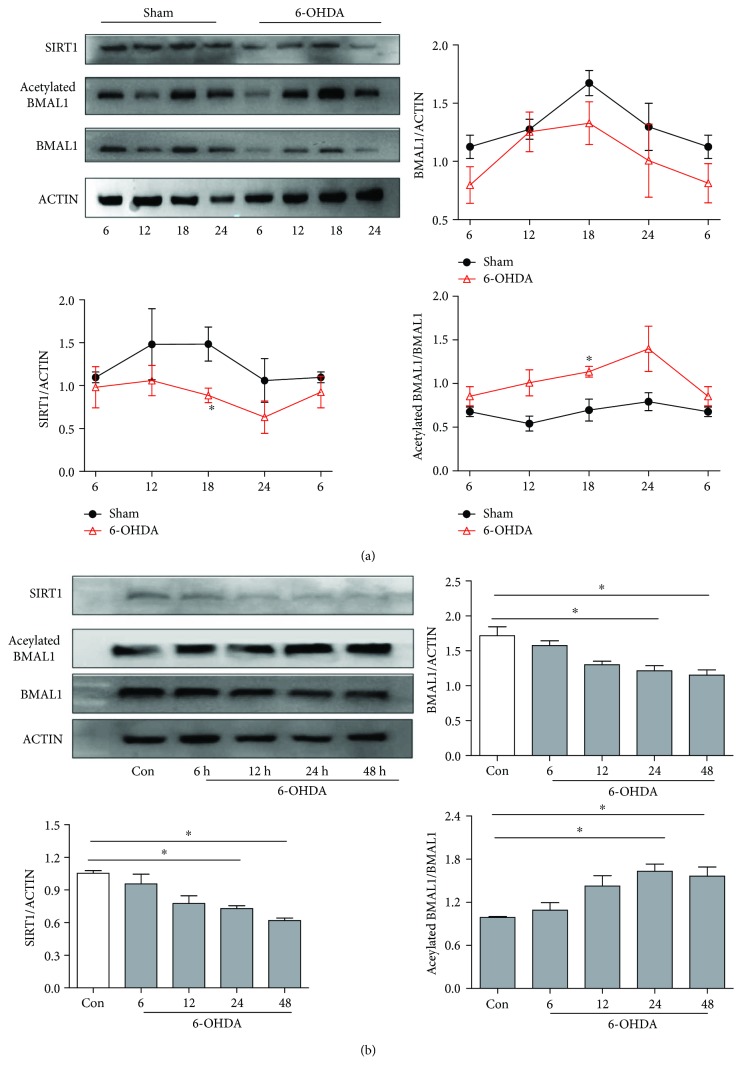
Acetylated BMAL1 was elevated, and SIRT1 was decreased in 6-OHDA-treated rats and SH-SY5Y cells. (a) Expression of BMAL1, acetylated BMAL1, and SIRT1 in the striatum of 6-OHDA-lesioned rats. (b) Expression of BMAL1, acetylated BMAL1, and SIRT1 in 6-OHDA-treated SH-SY5Y cells. ^∗^*P* < 0.05 versus the control group.

To investigate the mechanism of the 6-OHDA-induced disruption of antioxidative activity, we measured levels of SIRT1 and acetylated BMAL1 after 6-OHDA treatment. In the striatum of sham rats, protein levels of SIRT1 and BMAL1 displayed circadian rhythms, with a peak at 18:00 (Figure 4(a)). However, 6-OHDA reduced levels of SIRT1 and BMAL1 throughout most of the day. As SIRT1 could modulate the circadian clock by regulating the acetylation of BMAL1, we also measured levels of acetylated BMAL1. We found that 6-OHDA increased the acetylation of BMAL1, perhaps as a result of the reduced SIRT1 level. In line with these findings, 6-OHDA-treated SH-SY5Y cells showed time-dependent reductions in BMAL1 and SIRT1 protein levels as well as an increased level of acetylated BMAL1 (Figure 4(b)).

### 3.5. Rescue of Clock and Antioxidative Gene Expression by a SIRT1 Agonist

**Figure 5:**
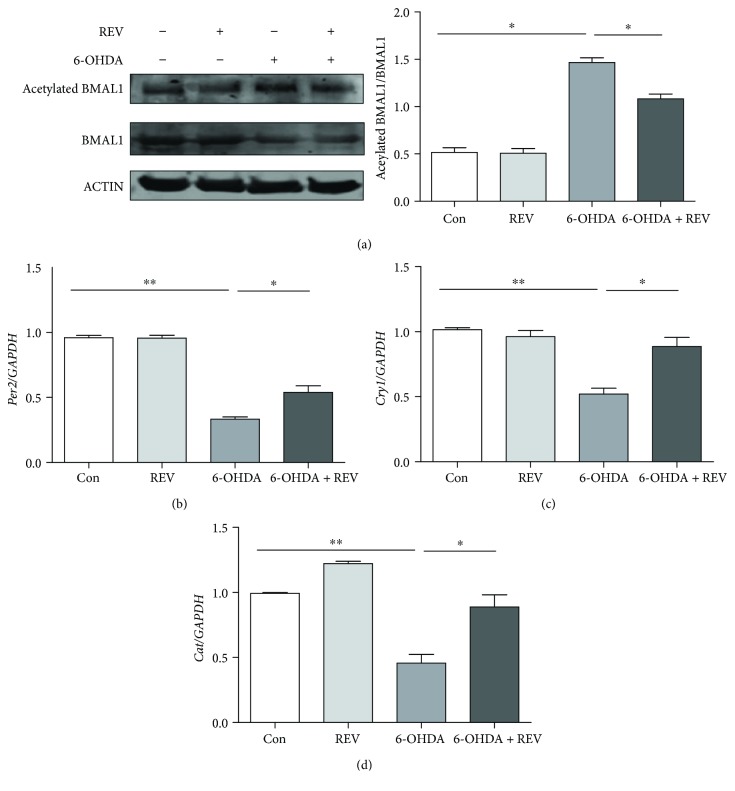
SIRT1 agonist rescued clock and antioxidative gene expression after 6-OHDA treatment. (a) Expression of BMAL1 and acetylated BMAL1. (b) Expression of *Per2*. (c) Expression of *Cry1*. (d) Expression of *Cat*. ^∗^*P* < 0.05, ^∗∗^*P* < 0.01.

To confirm the role of the SIRT1-BMAL1 pathway in 6-OHDA-induced disruption of circadian rhythm and antioxidant activity, we treated SH-SY5Y cells with 50 *μ*M the SIRT1 agonist resveratrol [23]. Pretreatment with resveratrol for 12 h partially reversed the increased level of acetylated BMAL1 in 6-OHDA-treated cells (Figure 5(a)). The decreased mRNA levels of clock genes *Per2* and *Cry1* were also partially rescued by resveratrol (Figures 5(b) and 5(c)), whereas *Clock* and *Bmal1* mRNA levels were unaffected (data not shown). Furthermore, resveratrol increased the mRNA level of the antioxidative gene *Cat* (Figure 5(d)).

### 3.6. Increased BMAL1-CRY1 Binding in 6-OHDA-Treated SH-SY5Y Cells

**Figure 6:**
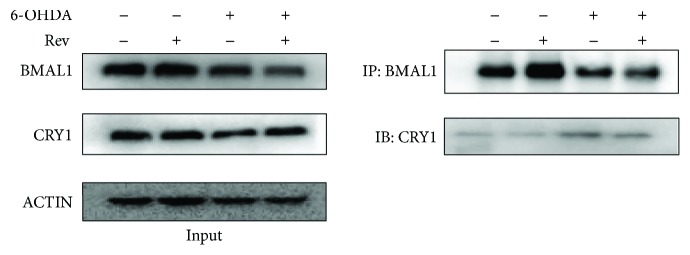
BMAL1 and CRY1 binding was increased after 6-OHDA treatment, which was reversed by SIRT1 agonist.

To determine whether 6-OHDA decreases downstream gene expression by enhancing the binding of BMAL1 and CRY1, we performed coimmunoprecipitation experiments. We found that 6-OHDA decreased BMAL1 and CRY1 protein levels in SH-SY5Y cells (Figure 6). However, BMAL1-CRY1 binding was increased in 6-OHDA-treated cells compared with control cells. After resveratrol pretreatment, the amount of CRY1 pulled down by a BMAL1 antibody decreased, suggesting that resveratrol reduces BMAL1 and CRY1.

## 4. Discussion

Patients with PD often experience circadian rhythm disruption, although normal biorhythms are essential for regulating antioxidative function. Oxidative stress is an important pathological mechanism of PD, but how circadian dysfunction affects antioxidative activity is not well known, especially in PD. Here, we found that 6-OHDA decreased the expression of circadian clock and antioxidative genes *in vitro* and *in vivo*. 6-OHDA also decreased levels of SIRT1, which may be related to an observed increase in acetylated BMAL1. A SIRT1 agonist reversed the 6-OHDA-induced increase in acetylated BMAL1 level and enhanced the expression of clock and antioxidative genes. Furthermore, 6-OHDA promoted the binding of BMAL1 and CRY1, which could weaken the positive effect of BMAL1 on target gene expression, and this binding was partially inhibited by a SIRT1 agonist. Therefore, our findings indicate that the SIRT1-BMAL1 pathway may contribute to abnormal antioxidative activity resulting from circadian rhythm dysfunction in PD.

Evidence of circadian rhythm dysfunction, such as sleep disorders and abnormal daily patterns of blood pressure and body temperature, is commonly found in the majority of PD patients [2, 24]. At the molecular level, Clock genes are also disturbed in PD patients and animal models of PD [3, 25]. Consistent with these previous studies, we found that *Bmal1*, *Per2*, and *Clock* expressions were down-regulated in the striatum of 6-OHDA-lesioned rats. There are several possible explanations for these changes in clock gene expression. First, dopamine receptor agonists can affect the expression of several clock genes, such as *Clock* and *Per*, in a receptor-dependent manner [26]. Moreover, dopamine could up-regulate *Per2* expression by elevating the transcriptional activity of the CLOCK-BMAL1 complex [27]. Second, suprachiasmatic nucleus (SCN) neurons exhibit reduced spontaneous firing rates in PD models, which may disturb the expression of clock genes in other brain regions and peripheral tissues [4]. Third, physical activity is a critical zeitgeber for the SCN. Altered rest-activity patterns and decreased levels of daytime activity are common phenomena in PD. In our study, the motor deficit was also found after 6-OHDA lesion, which may result in reduced signal input for the SCN. Fourth, there are interconnections between the oxidative homeostasis and circadian rhythm, and the abnormal production of ROS could inversely affect the circadian function [28]. In addition, ROR*α* is thought to positively regulate the expression of target genes such as *Bmal1* [29]. Thus, our observation of increased *Rorα* expression in our PD model suggests that this may be a compensatory response to a low level of BMAL1.

Increased oxidative stress and a loss of antioxidative defense are critically involved in the onset of PD [30]. Therefore, improving antioxidative function is a promising avenue for PD therapy. In the present study, we observed impaired antioxidative activity with low levels of related enzymes in 6-OHDA-lesioned rats. Along with this, the ROS and LPO products were increased in the PD model. Interestingly, several studies show that the expression and activity of antioxidative molecules including CAT, SOD, GPx, and GST exhibit daily rhythms, indicating that they are controlled by the endogenous biological clock [8]. Most clock-controlled genes are regulated through their E-box element, and base analysis shows that GPx, CAT, and GST also have classical E-box and E-box-like elements in their promoter regions. What is more, circadian genes are related with endoplasmic reticulum stress and play a critical role in sustaining the homeostasis balance. ROS production and scavenging are under control of diurnal cycles; thus, the organism could coordinate the metabolic process [31]. Oppositely, the abnormal expression of clock genes could result in the oxidative stress and ultimately lead to cell damage and senescence [11]. In addition, therefore, the attenuated antioxidative function observed in 6-OHDA-lesioned rats may be partially due to disrupted circadian rhythm.

Along with altered antioxidative activity, we observed a reduced level of SIRT1, which is in line with previous studies [19]. SIRT1 exerts protective effects against PD via several pathways, including anti-inflammation, antioxidative stress, and up-regulation of autophagy [32]. Moreover, SIRT1 is closely related to biorhythms and plays an important role in the deacetylation of BMAL1 [21]. Thus, a reduced level of SIRT1 can lead to a high acetylation ratio of BMAL1, which may alter the conformation and affect the function of BMAL1. Consistently, we detected elevated acetylation of BMAL1 after 6-OHDA treatment *in vitro* and *in vivo*, which was reversed by a SIRT1 agonist. On the other hand, CLOCK could acetylate BMAL1, which could increase the recruitment of CRY1 to the CLOCK-BMAL1 complex and facilitate repression of CLOCK-BMAL1-dependent transcription [33]. Although SIRT1 also promotes the deacetylation and degradation of PER2 [20], we found decreased PER2 expression after 6-OHDA treatment, suggesting that the positive effect of the BMAL1-CLOCK complex on PER2 might be stronger than the negative effect of SIRT1 on the BMAL1-CLOCK complex. Also, our coimmunoprecipitation experiments suggest that 6-OHDA promoted the binding of BMAL1 and CRY1, which was reversed by a SIRT1 agonist. With increased BMAL1-CRY1 binding, the BMAL1-CLOCK complex may bind less to the E-box elements of downstream genes, which could attenuate its positive regulatory effect and reduce the expression of target genes. Furthermore, we found that the level of SIRT1 showed circadian rhythms, and a previous study shows the existence of two E-box elements in the promoter region of SIRT1, similar to other clock-targeted genes [34]. Therefore, a high acetylated BMAL1 level could negatively affect the level of SIRT1.

In conclusion, our results show that 6-OHDA suppressed the expression of *Bmal1*, *Clock*, and *Per2* and altered circadian rhythms of antioxidative gene expression in *in vivo* and *in vitro* PD models. Moreover, a SIRT1 agonist attenuated 6-OHDA-mediated circadian rhythm dysfunction and abnormal antioxidative activity, suggesting that these pathologies are mediated by the SIRT1-BMAL1 pathway. Thus, agents regulating the molecular circadian clock could be a novel therapy for slowing the progression of PD.

## Abbreviations

PD: Parkinson's disease
6-OHDA: 6-Hydroxydopamine
SIRT1: Silent information regulator 1
BMAL1: Brain and muscle Arnt-like protein 1
PER: Period circadian protein
CRY: Cryptochrome
CAT: Catalase
SOD: Superoxide dismutase
GPx: Glutathione peroxidase
GSH: Glutathione
ROR*α*: Retinoid acid receptor-related orphan receptor *α*
GST: Glutathione S transferase
SCN: Suprachiasmatic nucleus
LPO: Lipid peroxide
ROS: Reactive oxygen species.
